# Stretchable Fiber‐Based Organic Light‐Emitting Diodes Display Enabled by Robust, Conductive, and Deformable Via

**DOI:** 10.1002/advs.202514594

**Published:** 2025-10-07

**Authors:** Yong Ha Hwang, Hagseon Kim, Kyung Cheol Choi

**Affiliations:** ^1^ School of Electrical Engineering Korea Advanced Institute of Science and Technology Daejeon 34141 Republic of Korea

**Keywords:** fiber displays, fiber OLEDs, stretchable displays, textile displays

## Abstract

Organic light‐emitting diode (OLED)‐based fiber displays emerge as promising candidates for next‐generation fiber display technologies, owing to their excellent optical performance and strong potential, as OLEDs are the mainstream technology in the display industry. However, despite these advantages, achieving stretchability—an intrinsic characteristic of textiles and an essential requirement for wearability—has remained a significant challenge. This limitation stems from the lack of strategies to resolve the inherently conflicting challenge of sustaining stable electrical interconnections between data and scan lines under mechanical deformation, such as stretching, within an *x–y* matrix architecture. Here, the first stretchable fiber‐based OLED display is reported, enabled by a robust, conductive, and deformable vertical interconnect access (via) that ensures stable operation under stretching‐induced mechanical strain. The novel via, fabricated from a conductive elastomer, simultaneously exhibits electrical conductivity, flexibility, and strong chemical bonds with interconnect lines. Furthermore, the pillar‐like structure is specifically designed to relieve mechanical stress, thereby achieving optimal deformability and improved structural robustness, resulting in only a ≈2.5% increase in resistance and a ≈7.1% decrease in luminance under 60° rotational strain. System‐level evaluations demonstrate reliable operation under ≈22.5% stretching, representing a critical breakthrough toward the practical realization of truly wearable fiber OLED displays.

## Graphical abstract

A stretchable OLED fiber display is developed by introducing a novel robust, conductive, and deformable via (RCD‐via) that maintains electrical and mechanical integrity under strain. Through scalable integration and mechanical design, the display operates reliably even during stretching, offering a promising strategy for truly wearable textile electronics.

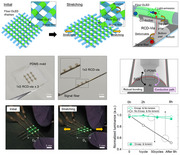

## 1 Introduction

Display technologies have progressively evolved toward user‐centric form factors, as demonstrated by the shift from stationary TVs to handheld smartphones and wearable devices like smartwatches.^[^ ^1^, ^2^, ^3^, ^4^ ^]^ In this paradigm shift, organic light‐emitting diode (OLED) technology has emerged as the mainstream display solution, owing to its exceptional optical performance, thinness, and flexibility.^[^ ^5^, ^6^ ^]^ These attributes have allowed OLEDs to lead the development of new free‐form factors, including foldable, rollable, wearable, and stretchable displays.^[^ ^7^, ^8^ ^]^ Meanwhile, fiber‐based displays have garnered growing interest as a next‐generation form factor for wearable displays, as, unlike conventional accessory‐type wearables, they can be directly woven into textiles—enabling truly wearable, garment‐integrated displays that ultimately enhance user convenience and accessibility.^[^ ^9^, ^10^, ^11^, ^12^, ^13^, ^14^, ^15^, ^16^ ^]^ To realize the ultimate goal, various light‐emitting devices such as alternating current electroluminescence (ACEL),^[^ ^17^, ^18^, ^19^, ^20^ ^]^ light‐emitting electrochemical cells,^[^ ^21^ ^]^ inorganic light‐emitting diodes (LED),^[^ ^22^, ^23^, ^24^ ^]^ and OLEDs have been explored at both the device and display levels.^[^ ^25^, ^26^, ^27^, ^28^, ^29^, ^30^, ^31^ ^]^ Among them, ACEL‐based fiber displays have shown outstanding breakthroughs in stretchability, enabling *x–y* matrix‐woven textile displays to elongate seamlessly—much like how garments stretch when pulled.^[^ ^20^ ^]^ This is because, unlike OLEDs or LEDs, the electroluminescence in ACEL devices does not require a physical junction at the crossing points; instead, light emission occurs across the entire area under an alternating electric field, allowing the intersecting fibers to remain mechanically decoupled.^[^ ^20^, ^32^ ^]^ By fully realizing these intrinsic characteristics of textiles, ACEL‐based fiber displays have enabled the practical implementation of fiber displays capable of presenting information even while being worn on the body. However, due to intrinsic limitations of the ACEL light source—such as high driving voltage, low current efficiency, and the lack of transistor compatibility that can provide high‐quality displaying—ACEL fiber displays still suffer from low‐performance operation. Meanwhile, OLED‐based fiber displays have demonstrated promising strategies to achieve electroluminescent performance comparable to that of conventional OLEDs at the individual device level, and have even been extended to system‐level addressable displays.^[^ ^25^, ^28^, ^31^ ^]^ Nevertheless, due to the structural and fabrication complexity of OLEDs, these fiber displays have struggled to achieve the essential characteristic of the textile form factor—particularly stretchability—that ACEL‐based fiber displays have demonstrated. One of the most critical bottlenecks preventing the practical implementation of stretchable OLED fiber displays lies in their inability to maintain reliable operation under mechanical deformation. In particular, although OLED displays based on *x–y* matrix architectures require vertical interconnect access (via) to electrically connect scan and data lines,^[^ ^33^ ^]^ no existing structural strategy for vias has successfully ensured signal reliability under stretching conditions.^[^ ^28^, ^31^, ^34^, ^35^ ^]^ This is primarily because, to achieve reliable operation under stretchable motion, three key features that are intrinsically conflicting must be simultaneously realized in the interconnection structure: 1) electrical conductivity for signal transmission between the scan and data lines, 2) mechanical deformability to accommodate stretching, and 3) robust adhesion to both scan and data lines to resist stress‐induced delamination. In previous studies, fiber‐based OLED displays have been developed either without incorporating vias or by connecting scan and data lines using simple silver paste; however, reliable stretchable performance has yet to be demonstrated.

Here, the first stretchable fiber‐based OLED display is reported, enabled by a novel robust, conductive, and deformable via (RCD‐via) structure that simultaneously fulfills all three conflicting requirements. The RCD‐via is composed of a PDMS elastomer embedded with silver microparticles (Ag MPs), providing both conductivity and mechanical softness. Our consideration is particularly important, as it enables the novel via to remain strongly anchored to both scan and data lines even under stretching, while also ensuring conductivity and mechanical softness. This is achieved through APTES (3‐aminopropyl) triethoxysilane and plasma treatment, which facilitates strong siloxane chemical bonding between the via's PDMS matrix and the metal contacts, while also ensuring electrical connectivity between the embedded silver microparticles (Ag MPs) and the metal lines. Furthermore, to prevent structural yielding (i.e., irreversible deformation or failure of the material) under stretching, the novel via structure is mechanically engineered based on governing equations derived from four boundary conditions—including the top and bottom pads oriented in intersecting directions and the connecting pillar between them—allowing the optimal pillar height and thickness to be determined. Notably, the unique structure comprising the top pad, bottom pad, and connecting pillar reduces stress concentration during stretching by ≈15% compared to conventional pillar designs, thereby enabling mechanically stable deformation. Due to the careful design, the RCD‐via allows the OLED‐based fiber display to operate stably even under 60° of rotational deformation, with only a ≈2.5% increase in resistance and a ≈7.1% decrease in luminance. In addition, to verify scalability, a 3 × 3 matrix of RCD‐vias was fabricated to connect three scan and three data lines to each other. It is noteworthy that the resulting 3 × 3 fiber OLED display exhibited stable operation under ≈22.5% stretching, establishing a crucial breakthrough toward practical, wearable, and stretchable OLED fiber displays.

## 2 Results and Discussion

**Figure 1:**
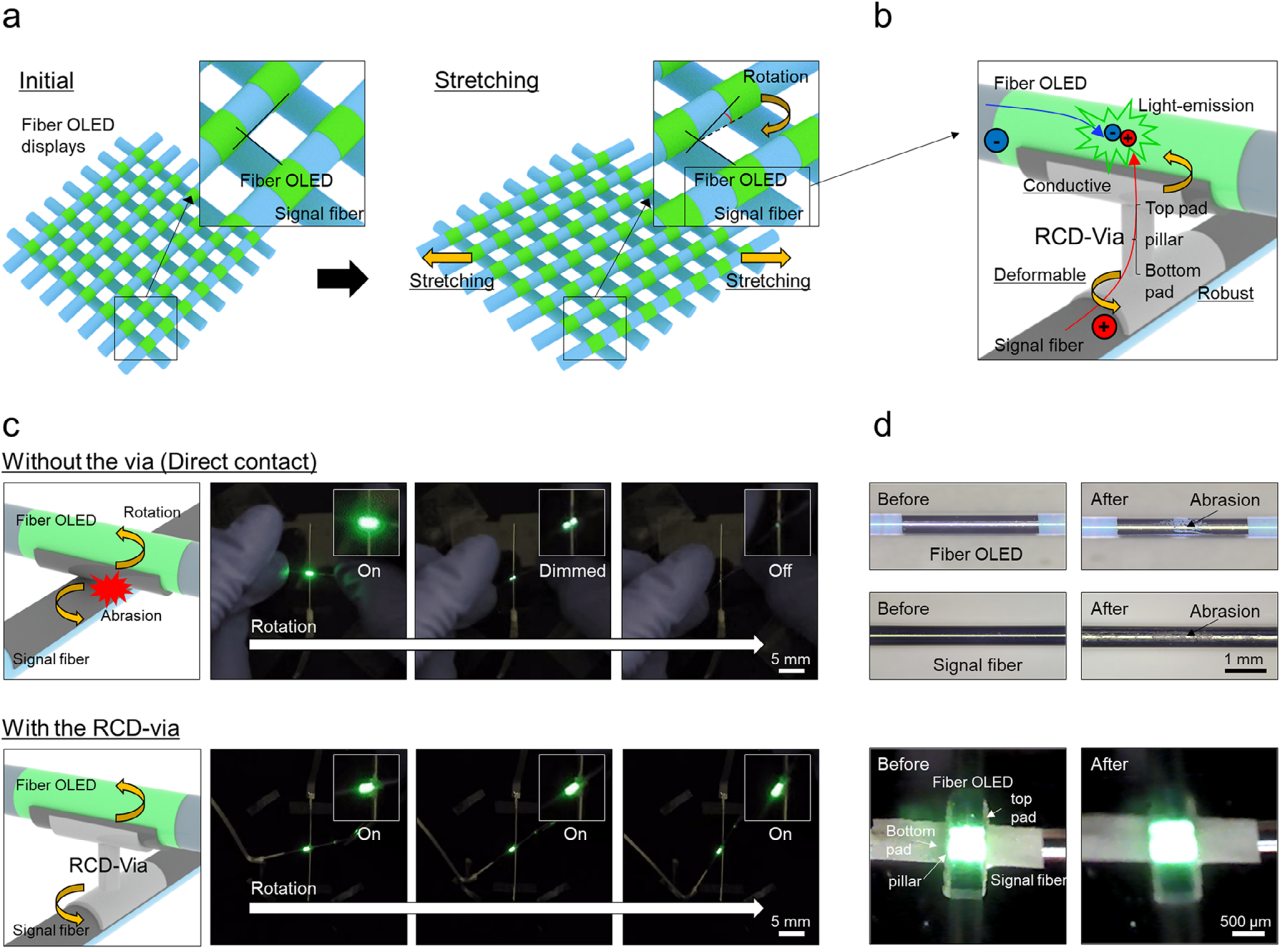
a) Schematic illustration of rotational deformation occurring at intersection points in an *x–y* matrix‐configured fiber OLED display under stretching. b) Operational mechanism of an OLED pixel driven through an RCD‐via at an intersection point. c,d) Comparison of device stability under rotation deformation with and without the RCD‐via, including optical microscopy images showing damage in the absence of the via.

**Figure** **1**a illustrates the deformation behavior of a fiber‐based OLED display constructed in an *x–y* matrix configuration, where scan lines (*x*‐direction) and data lines (*y*‐direction) are electrically connected at each intersection point to drive the OLED pixels. Upon mechanical stretching, rotational displacement occurs at these intersections, highlighting the necessity of an interconnection structure that can accommodate such rotational motion while ensuring reliable signal transmission. Figure 1b provides a more detailed explanation of how a single pixel is operated at the intersection of an *x–y* matrix when the via is present. Hole injection occurs from the signal fiber's metal layer (Aluminum (Al)), passes through the via, and reaches the OLED anode (Al). At the same time, electrons are injected from the data line side, traverse the cathode (PEDOT: PSS), and recombine with holes in the emissive layer, resulting in light emission from the top surface of the fiber OLED (Figure S1, Supporting Information). Therefore, as previously discussed, the interconnection at the intersection must simultaneously satisfy three essential requirements: it must be conductive to ensure signal transmission, robustly bonded to each Al line for stable electrical contact under stretching, and mechanically deformable to accommodate rotational strain. To fulfill these criteria, a robust, conductive, and deformable via (RCD‐via) is introduced, specifically engineered to meet all three conflicting requirements. The RCD‐via is composed of polydimethylsiloxane (PDMS) mixed with 83 wt.% silver microparticles (Ag MPs), providing both electrical conductivity and mechanical flexibility.^[^ ^36^ ^]^ To accommodate rotational strain, the novel via—mechanically designed with a top pad, a vertically aligned pillar, and a bottom pad—is introduced, the design of which will be discussed in detail in a later section. The top and bottom pads are rectangular in shape and are in contact with the upper and lower fibers with the Al film, respectively. As a result, when viewed from the top, the overall structure forms a cross shape rotated by 90°. Each pad is chemically bonded to the respective Al line on the signal and OLED fiber via a combination of APTES (3‐aminopropyltriethoxysilane) activation and oxygen plasma treatment, ensuring strong mechanical adhesion and continuous electrical conduction. The detailed design rationale is also further discussed in later sections (Figure S2, Supporting Information). To validate the necessity of the RCD‐via, Figure 1c,d compares the operational stability of OLED devices under rotational strain with and without the RCD‐via. In the absence of the via, signal transfer occurs through direct contact between the Al layers of the signal and OLED fibers. Under rotational deformation, this contact becomes unstable due to interfacial friction, leading to mechanical damage of the thin films and resulting in degraded OLED operation. This physical damage is corroborated by optical microscopy images. In contrast, devices incorporating the RCD‐via maintain stable OLED operation even under rotation, clearly demonstrating the via's effectiveness in preserving both mechanical and electrical integrity (Movie S1, Supporting Information).

**Figure 2:**
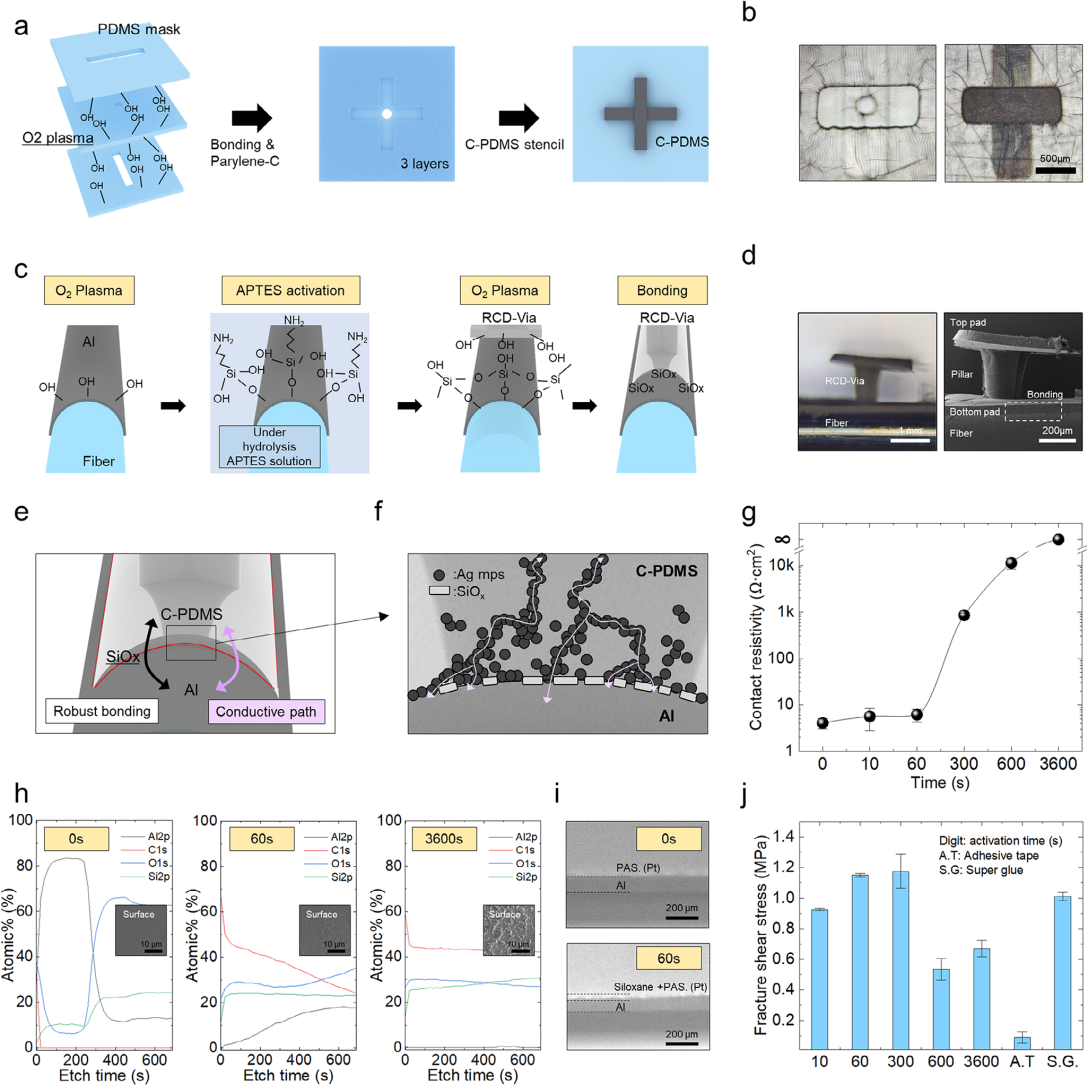
a) Schematic of the RCD‐via fabrication process using a three‐layer PDMS mold and stencil filling of C‐PDMS. b) Optical image of the PDMS mold and top view of a completed RCD‐via. c) Chemical bonding mechanism and process between the RCD‐via and Al surfaces via APTES‐assisted siloxane bonding. d) OM image showing successful bonding of the RCD‐via onto a fiber surface. e) Schematic of the dual‐mode bonding mechanism achieving both robustness and electrical conductivity. f) XPS analysis showing the trade‐off between conductivity and bonding strength depending on APTES activation time. g) Contact resistance versus APTES activation time. h) XPS analysis showing the atomic ratios of Al, C, O, and Si. Inset: Optical microscopy and XPS images showing surface changes over time. i) FIB cross‐sectional image showing siloxane bonding layer formation. j) Fracture shear stress between the RCD‐via and Al thin film at various APTES activation times.

**Figure** **2**a illustrates the fabrication process of the RCD‐via, which consists of the bottom pad, the vertically aligned pillar, and the top pad, as previously described. Accordingly, a three‐layer PDMS mold capable of defining these distinct structures was fabricated, and a stencil method was employed to construct the RCD‐via. To fabricate each PDMS mold, after PDMS curing, laser patterning is used to create individual PDMS molds for each of the three patterns. Subsequently, oxygen plasma treatment is applied to the bonding surfaces of each mold to generate hydroxyl groups (─OH), as the silane functional groups (─(CH_3_)_2_Si─) on the PDMS surface are converted to silanol (Si─OH) groups. When these surfaces are brought into physical contact, strong covalent siloxane bonds (Si─O─Si) are formed, resulting in a single integrated three‐layer mold.^[^ ^37^ ^]^ To prevent mixing with the conductive PDMS composite (C‐PDMS), composed of PDMS and 83 wt.% silver microparticles (Ag MPs), a 200 nm layer of parylene‐C is deposited on the surface of the PDMS mold prior to stencil printing and curing.^[^ ^38^ ^]^ Without this layer, the C‐PDMS would mix with the PDMS mold during curing. C‐PDMS is then filled into the assembled PDMS mold using a stencil method and cured to form the RCD‐via (Figure S3, Supporting Information). Figure 2b provides a top‐view optical microscope (OM) image of the completed PDMS mold, along with the RCD‐via structure formed by stencil‐filling C‐PDMS into the patterned cavities. Figure 2c explains the bonding mechanism and process that enables robust attachment of the RCD‐via to the fiber's Al metal layer. Initially, oxygen plasma treatment is used to introduce hydroxyl groups onto the Al surface. Following this, the fiber is immersed in an APTES solution (EtOH:DI: APTES = 90:5:5 (v/v/v)), where hydrolysis leads to the formation of silanol groups capable of forming siloxane bonds with the hydroxylated Al surface.^[^ ^39^ ^]^ A subsequent oxygen plasma treatment on the PDMS surface of the RCD‐via's pad generates additional ─OH groups, and bringing the surfaces into contact facilitates the formation of strong siloxane bonds. Figure 2d presents an optical microscope image, in which the RCD‐via is clearly shown to be successfully bonded to the fiber surface. Figure 2e,f details the dual‐functional bonding mechanism that allows the RCD‐via to achieve both robustness and electrical conductivity. In this configuration, a portion of the contact area (PDMS) forms strong mechanical bonds via siloxane linkages, while the remainder enables electrical conduction through physical contact between Ag MPs and the Al surface. The trade‐off between these properties is determined by the APTES activation time. If the activation time is too short, insufficient siloxane bonding sites are formed, resulting in good conductivity but weak mechanical adhesion. Conversely, longer activation times lead to increased SiOx accumulation, which enhances bonding strength but degrades conductivity. To experimentally determine the optimal condition, the change in contact resistivity was measured as a function of APTES activation time. As shown in Figure 2g, minimal change was observed up to 60 s, but a significant increase occurred beyond this point. To quantify this effect, X‐ray photoelectron spectroscopy (XPS) analysis was performed at varying APTES activation times (Figure 2h). As shown in the results, Al peak intensity decreases while Si peak intensity increases with longer activation times (0 to 60 to 3600 s), indicating the progressive formation of SiOx and byproducts on the surface. Inset optical microscope images also verify this trend, showing a clean Al surface at 0 s, slight surface spotting at 60 s, and substantial byproduct accumulation at 3600 s. Additional intermediate conditions, including 10, 300, and 600 s activation, are presented in Figure S4 (Supporting Information). Focused ion beam (FIB) cross‐sectional imaging also supports the formation of a siloxane bonding layer at 60 s (Figure 2i). Following the contact resistance results, to evaluate bonding strength, shear tests were conducted to measure fracture shear stress between the RCD‐via and Al thin film at various APTES activation times (Figure 2j) (Figure S5, Supporting Information). The highest fracture shear stress was observed between 60 and 300 s, comparable to that of commercial superglue, due to the optimal number of siloxane bonding sites. However, beyond 300 s, excessive siloxane formation and byproducts reduced bonding performance. Based on these results, an APTES activation time of 60 s was selected as the optimal condition, achieving both low contact resistance and high bonding strength.

**Figure 3:**
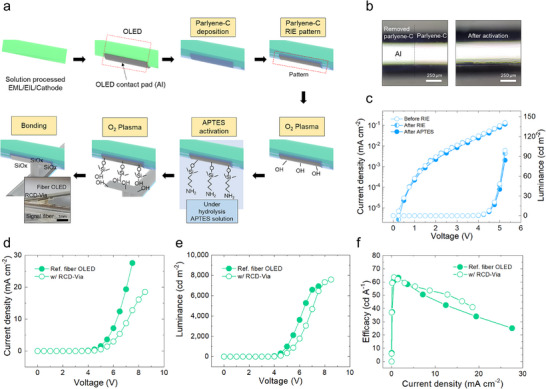
a) Schematic of the fiber OLED structure and the top pad bonding process. b) Selective patterning of parylene‐C using a PDMS mask and RIE to expose the Al top contact. c) *J–V–L* characteristics before and after RIE and APTES activation, showing stable device performance. d–f) Comparison of *J–V–L* curves for OLEDs with and without the RCD‐via, showing a ≈1 V voltage drop due to via resistance.

**Figure** **3**a shows the bonding process of the RCD‐via top pad onto the anode (Al) of the fiber OLED. To apply the solution‐processed fiber OLED structure reported in previous work, layers from the cathode to the emissive layer (EML) are uniformly deposited over the entire surface of the cylindrical fiber. Then, the electron transport layers and anode layers are selectively deposited on one half of the fiber using thermal evaporation. A 500 nm layer of parylene‐C is subsequently deposited over the full cylindrical surface via chemical vapor deposition (CVD). Using a PDMS mask, all areas except the top of the Al layer are covered. Reactive ion etching (RIE) is then performed to selectively remove the parylene‐C from the exposed Al surface, forming a clean bonding pattern. As shown in Figure 3b, the regions still covered with parylene‐C appear slightly pinkish compared to the patterned areas where the parylene‐C has been removed. Following this, similar to the bonding process between the signal fiber and the RCD‐via bottom pad, oxygen plasma treatment and APTES activation are applied to form siloxane bonding. This ensures both robustness and electrical conductivity. To validate the reliability of each process step, the OLED current density–voltage–luminance (*J–V–L*) characteristics were compared before and after RIE and APTES activation, showing no significant performance degradation, as presented in Figure 3c. Figure 3d–f presents a comparison of the *J–V–L* curves for fiber OLEDs driven directly and those driven through the RCD‐via. Due to the intrinsic resistance of the C‐PDMS material, composed of PDMS and Ag MPs, the RCD‐via‐connected device exhibits a voltage drop of ≈1 V, though this does not significantly impair OLED operation, and overall performance remains comparable.

**Figure 4:**
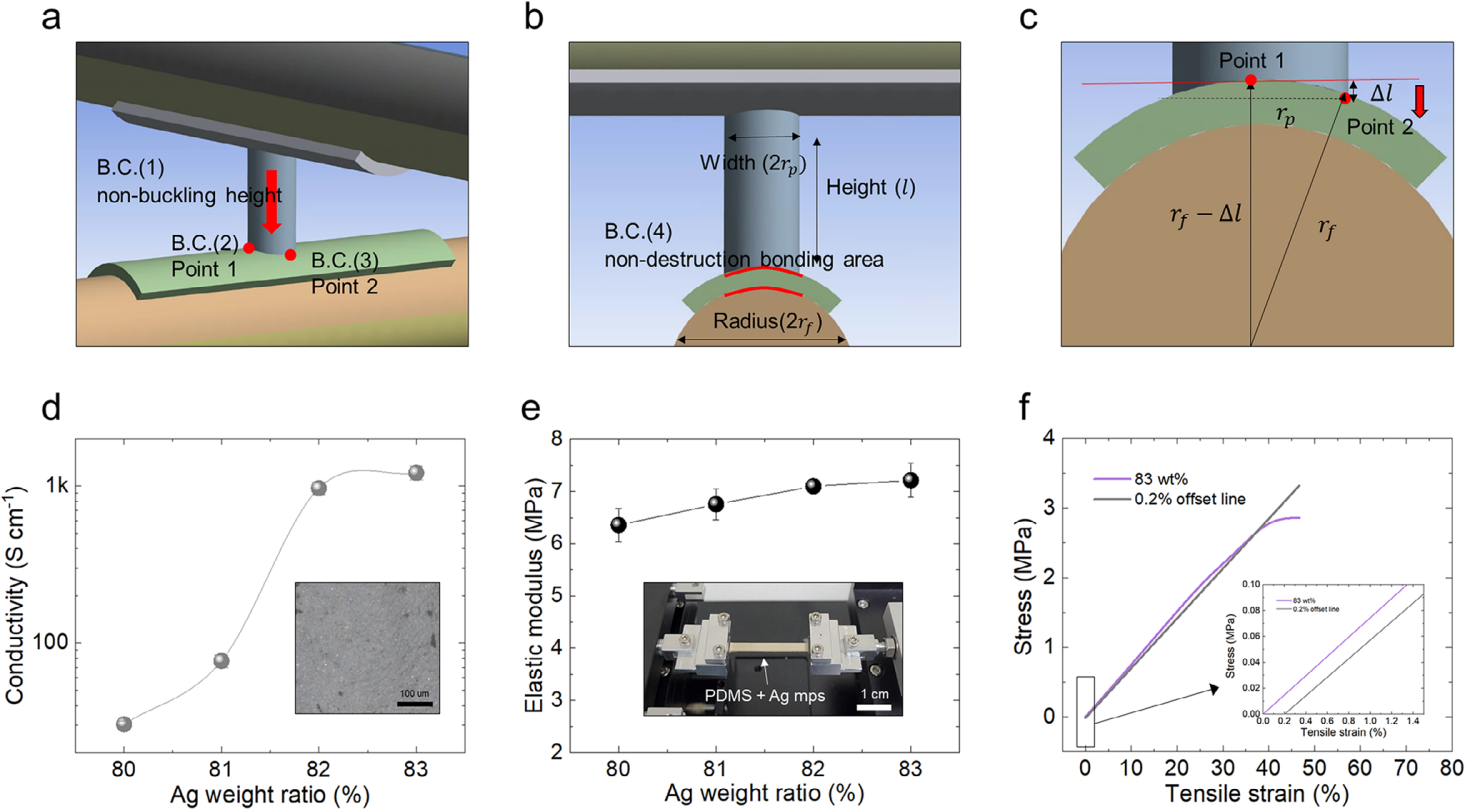
a–c) Four mechanical boundary conditions considered in via design: critical slenderness ratio, torsional stress at two points, and contact curvature. d) Electrical conductivity of C‐PDMS as a function of Ag microparticle content. e) Tensile modulus of C‐PDMS at different compositions. f) Stress–strain curve of optimized C‐PDMS (83 wt.%), indicating yield strength.

**Figure** **4**a–c shows the four boundary conditions established to determine the appropriate height and width of the RCD‐via required to withstand strain during stretching without failure. First, as illustrated in Figure 4a, the RCD‐via connecting the lower signal fiber and the upper fiber OLED is subjected to mechanical loading from the upper fiber OLED. Therefore, a critical slenderness ratio is required to prevent structural failure under this load (Boundary condition 1). Pillar structures are typically categorized as either short or long columns depending on this critical slenderness ratio, and buckling occurs primarily in long columns. To avoid buckling‐induced failure, the slenderness ratio must remain below the critical value, leading to the formulation of the first boundary condition.

(1) $$\lambda \;\;=\frac{l}{K}=\frac{l}{\sqrt{\frac{I}{A}}}=\frac{l}{\sqrt{\frac{\pi {\left(2r_{p}\right)}^{4}}{64}\times \frac{4}{\pi {\left(2r_{p}\right)}^{2}}}}=\frac{4l}{2r_{p}}=\frac{2l}{r_{p}}$$

where *λ* is the slenderness ratio, *r*_p_ is the radius of the pillar, *I* is the minimum area moment of inertia, and *A* is the cross‐sectional area of the pillar.^[^ ^40^ ^]^

(2) $$\;\lambda_{c}=\sqrt{\frac{2n\pi^{2}E}{\sigma y\;}}=\sqrt{\frac{\pi^{2}E}{\sigma y\;}}$$

where λ_c_ is the critical slenderness ratio, n is the buckling factor (*n* = 1 for pinned to pinned conditions), *E* is the Young's modulus, and σ_y_ is the yield strength. To ensure structural safety, the actual slenderness ratio must be smaller than this critical slenderness ratio. (λ <  λ_*c*_) Therefore, the first governing equation based on the equation can be established as follows:

(3) $$\frac{2l}{r_{p}}<\sqrt{\frac{\pi^{2}E}{\sigma y\;}}$$

next, when rotational deformation occurs, the maximum torsional stress is concentrated at the furthest points from the center of rotation. As shown in Points 1 and 2, the yield condition is established using the Tresca yield criterion,^[^ ^41^ ^]^ which states that yielding occurs when the maximum shear stress exceeds half of the yield stress (Boundary conditions 2 and 3). At Point 1, the principal stresses were determined under the assumption of pure torsion. Since pure torsion implies no normal stress in the *x‐* and *y*‐directions, the stress components *σ*_x1_ and *σ*_y1_ are both zero. Therefore, the stress tensor can be expressed as:

(4) $${\left(\tilde{\sigma }\right)}_{1}=\;\;\left(\begin{array}{cc} \sigma_{x1} & \tau_{xy1}\\ \tau_{xy1} & \sigma_{y1} \end{array}\right)=\;\;\left(\begin{array}{cc} 0 & \tau_{xy1}\\ \tau_{xy1} & 0 \end{array}\right)$$

To determine the principal stresses and their directions, the stress tensor was analyzed using the eigenvalue problem:

(5) $$Determinant=0,\;\left|{\left(\tilde{\sigma }\right)}_{1}-\lambda \right|=\left|\begin{array}{cc} -\lambda & \tau_{xy1}\\ \tau_{xy1} & -\lambda \end{array}\right|,\;∴\lambda =\pm \tau_{xy1}$$

(6)

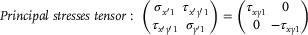




According to the Tresca yield criterion, yielding occurs when the maximum shear stress exceeds half of the yield stress. Therefore, to avoid yielding at Point 1, the condition $\frac{|\sigma_{1\;}-\;\sigma_{2}|}{2}=\tau_{xy1}<\tau_{max}$ must be satisfied. Based on this, the second governing equation at Point 1 can be established as follows:

(7) $$\tau_{xy1}=G*\gamma =\frac{E}{2\left(1+v\right)}*\;\frac{r_{p}\theta }{l}<\frac{\sigma_{y}}{2},$$

(8) $$∴\frac{E}{2\left(1+v\right)}*\;\frac{r_{p}\theta }{\sigma_{y}}<l$$

where *G* is the modulus of rigidity, γ is the shear strain, and ν is Poisson's ratio. Next, similar to Point 1, the same boundary condition must also be considered at Point 2. As shown in Figure 3c, Point 2 is located slightly below Point 1 by a distance of Δ*l* from the center of the pillar. To account for this offset, it is assumed that *σ*_y2_ = 0 and *σ*_x2_ = *E* × Δ*l*/*l*, representing the vertical stress induced at this point. Therefore, the stress tensor can be expressed as:

(9) $${\left(\tilde{\sigma }\right)}_{2}=\left(\begin{array}{cc} \sigma_{x2} & \tau_{xy2}\\ \tau_{xy2} & \sigma_{y2} \end{array}\right)=\left(\begin{array}{cc} \sigma_{x2} & \tau_{xy2}\\ \tau_{xy2} & 0 \end{array}\right)$$

To determine the principal stresses and their directions, the stress tensor was analyzed using the eigenvalue problem:

(10) $$Determinant=0,\;\left|{\left(\tilde{\sigma }\right)}_{2}-\lambda \right|=\left|\begin{array}{cc} \sigma_{x2}-\lambda & \tau_{xy2}\\ \tau_{xy2} & -\lambda \end{array}\right|,$$

(11) $$∴\lambda =\frac{\sigma_{x2}\pm \sqrt{{\sigma_{x2}}^{2}+4{\tau_{xy2}}^{2}}}{2}$$

(12)

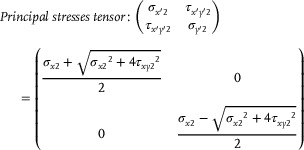




According to the Tresca yield criterion, the condition $\frac{|\sigma_{1}-\sigma_{2}|}{2}=\frac{\sqrt{{\sigma_{x2}}^{2}+4{\tau_{xy2}}^{2}}}{2}<\tau_{max}$ must be satisfied. Based on this, the third governing equation at Point 2 can be established as follows:

(13) $$\begin{array}{c} \sqrt{{\left(\;E\times \frac{\Delta l}{l}\;\right)}^{2}+4{\left(\;\frac{E}{2(1+v)}\times \frac{r_{p}\theta }{l+\Delta l}\;\right)}^{2}}<\sigma_{y}\Delta l=r_{f}-\sqrt{{r_{f}}^{2}-{r_{p}}^{2}} \end{array}$$

(14) $$\begin{array}{c} ∴\sqrt{{\left(\;E\;\times \;\frac{r_{f}\;-\;\sqrt{{r_{f}}^{2}\;-\;{r_{p}}^{2}}}{l}\;\right)}^{2}\;+\;4{\left(\;\frac{E}{2(1\;+\;v)}\;\times \;\frac{r_{p}\theta }{l\;+\;r_{f}\;-\;\sqrt{{r_{f}}^{2}\;-\;{r_{p}}^{2}}}\;\right)}^{2}}\;<\;\sigma_{y} \end{array}$$

The final, fourth boundary condition considers the deformation occurring at the curved contact surface, as observed in Figure 3b. To account for this curvature, the elastic deformation at the contact interface was estimated based on the indentation depth formula from nanoindentation theory, because the maximum elastic indentation was calculated for a rigid cylinder pressing onto a soft elastomeric surface.^[^ ^42^ ^]^

The indentation depth is given by:

(15) $$d=2r{\left(\frac{KH}{E}\right)}^{2},r_{p}=\sqrt{{\left(r_{f}\right)}^{2}-{\left(r_{f}-d\right)}^{2}}$$

(16) $$∴r_{p}=\sqrt{{\left(r_{f}\right)}^{2}-{\left(r_{f}-2r_{f}{\left(\frac{KH}{E}\right)}^{2}\right)}^{2}}$$

where *K* is the hardness coefficient, *H* is the hardness of the softer material, and *E* is the elastic modulus. This equation defines the final constraint on the allowable deformation at the curved contact surface to ensure elastic compliance without mechanical failure.

To experimentally determine the required material properties of C‐PDMS for satisfying the above four governing equations, mechanical tests were conducted. As shown in Figure 4d, the conductivity of C‐PDMS was evaluated as a function of Ag microparticle (Ag MPs) mixing ratio. The results indicated that the conductivity begins to saturate at ≈82 wt.%. Similarly, tensile testing results in Figure 4e show that the elastic modulus also begins to saturate at ≈82 wt.%. Based on these findings, a mixing ratio of 83 wt.% Ag MPs was selected as the optimal composition for C‐PDMS. At these conditions, the elastic modulus (*E*) was measured to be 7.122 MPa. To evaluate the yield strength (*σ*_y_), a stress–strain curve was obtained (Figure 4f), and the yield point was determined using the 0.2% offset method, yielding *σ*_y_ = 2.3 MPa. Finally, nanoindentation testing was performed to measure the hardness (*H*), which was found to be 2.23 MPa.

**Figure 5:**
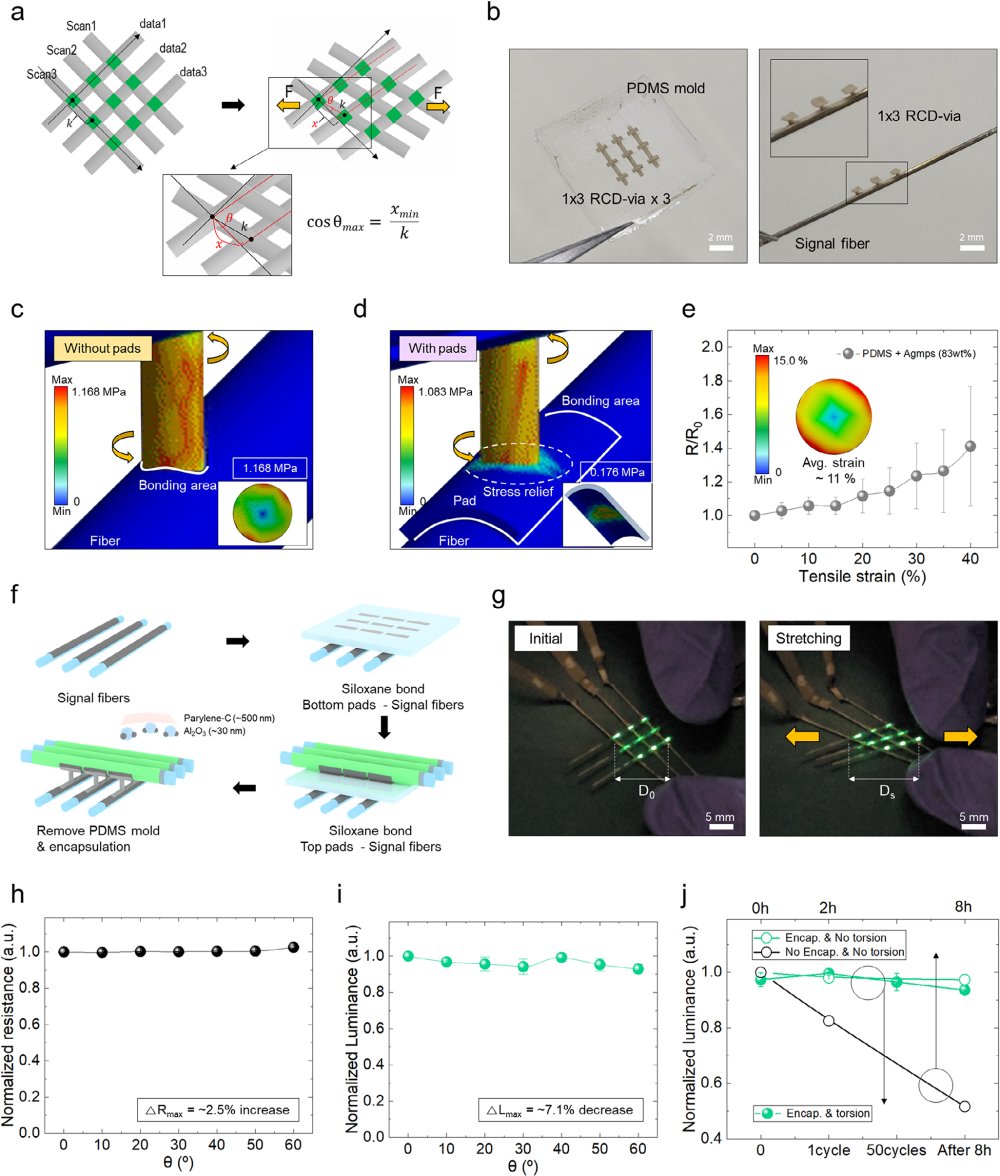
a) Maximum twist angle of the RCD‐via under fiber stretching conditions in a 3×3 matrix configuration. b) Optical image of a scalable 1 × 3 RCD‐via bonded to a signal fiber. c,d) FEA results comparing stress distribution in the RCD‐via with and without a base pad. e) Resistance change of the RCD‐via under tensile strain (inset: simulated strain). f) Fabrication sequence of the 3 × 3 OLED fiber display with orthogonal signal/data fiber arrangement and encapsulation. g) Photographs of the display before and after stretching (≈22.5% stretchability). h,i) Resistance and luminance variation under twisting angles, indicating minimal performance degradation. j) Luminance stability comparison between 1) encapsulated devices after 8 h, 2) encapsulated devices with and without 50 torsion cycles, and 3) non‐encapsulated devices after 8 h.

**Figure** **5**a illustrates the maximum rotational angle that the RCD‐via can experience at the intersection points of a 3 × 3 matrix OLED fiber display under stretching. Assuming a simple case where a gap equivalent to the diameter of a single fiber exists between two adjacent fibers aligned in the same direction, as typically seen in woven textiles, the initial spacing (or pitch) between fibers—defined as the center‐to‐center distance from one fiber to its adjacent fiber—can be approximated as 4r_p_. Under ideal full stretching, the fibers come into contact with each other, reducing the pitch to 2r_p_. Based on this geometric change, the maximum rotational angle during stretching can be estimated to be ≈60°. Based on this, using the four previously established boundary conditions and the measured material properties, the optimal pillar radius and height were determined to be ≈100 and 390 µm, respectively (Figure S6, Supporting Information). Figure 5b shows the scalable 1 × 3 RCD‐via structure fabricated in a PDMS mold and successfully bonded onto a signal fiber, demonstrating that the RCD‐via design is not limited to single‐pixel applications but can be extended to scalable m × n matrix‐type displays as a viable integration strategy. This scalable via consists of three vertically aligned pillars sharing a single bottom pad and individual top pads, which can be easily reconfigured by simply modifying the PDMS mold design during laser patterning. Figure 5c,d present finite element analysis (FEA) simulations of the mechanical stress experienced by the RCD‐via (with 100 µm radius and 390 µm height) under a 60° rotational strain, with and without the presence of pads, demonstrating that the designed structure is well‐suited to accommodate stretching both in terms of structural stress distribution and bonding integrity. First, in terms of structural stress distribution, in the case of the absence of the pad (Figure 5c), the pillar directly bonds to the relatively rigid PET surface with Al, resulting in a von Mises stress of up to 1.168 MPa. Conversely, when a base pad is included (Figure 5d), the stress is distributed across the top of the pad, reducing the von Mises stress by ≈8% to 1.083 MPa. This stress level is approximately half the measured yield strength, indicating sufficient mechanical safety under stretching. From a bonding perspective, the padless structure requires the bonding interface between the fiber and the pillar to withstand the full 1.168 MPa. However, with the inclusion of the pad, the bonding interface shifts to the base of the pad rather than the pillar. As a result, the bonding interface between the pad and the fiber only needs to withstand ≈15% of the stress seen in the padless structure (≈0.176 MPa), as calculated through simulation, which is well within the previously measured maximum shear strength of the siloxane bond formed between C‐PDMS and Al. Also, as shown in Figure 5e, the resistance change was measured under applied strain, and although the pillar experienced up to ≈11% strain, the C‐PDMS exhibited almost no change in resistance. This indicates that the display is expected to maintain nearly constant luminance and resistance even under stretching. This expectation is further validated by Figure 5h,i, which shows the resistance and luminance as functions of the stretching angle.

Meanwhile, Figure 5f illustrates the fabrication sequence of the 3 × 3 fiber OLED display. The scalable three RCD‐vias are first siloxane‐bonded onto three signal fibers, followed by siloxane bonding to the three patterned anodes of the three fiber OLEDs arranged in a direction orthogonal to the signal fibers. After physical removal of the PDMS mold, the entire 3D display structure is encapsulated with 30 nm of Al_2_O_3_ and 500 nm of parylene‐C via ALD and CVD, respectively. Figure 5g shows the functional 3 × 3 OLED fiber display before and after stretching. The system‐level stretching, representing the degree of stretching (*D*_s_/*D*_0_), was measured to be ≈22.5%, demonstrating reliable operation under stretching. (Movie S2, Supporting Information) To quantitatively evaluate operational stability under stretching, the variations in resistance and luminance as a function of the twisting angle experienced by the RCD‐via were tested. As a result, the resistance increased by ≈2.5%, and luminance decreased by ≈7.1%, indicating that display performance remains stable under stretching, as shown in Figure 5h,i. Furthermore, the proposed structure accommodates stretching solely through the RCD‐via region, enabling conventional vapor‐deposited encapsulation to effectively protect the OLED devices even under mechanical deformation. To demonstrate this, luminance variation was compared under three conditions: 1) an encapsulated device subjected to 50 torsion cycles, 2) an encapsulated device without torsion, and 3) a non‐encapsulated device (Figure 5j). While the non‐encapsulated device exhibited a 50% luminance decrease after 8 h due to oxidation, the encapsulated devices maintained stable brightness regardless of whether they underwent torsion or not. This result is noteworthy, as it indicates that the stretching occurs primarily in the RCD‐via region—not in the OLED device region—demonstrating that the OLED device areas remain stably encapsulated and protected from stretching motion. This thereby validates that even widely adopted vapor‐deposited encapsulation strategies can be effectively applied,^[^ ^43^, ^44^ ^]^ ultimately confirming the practical applicability of stretchable OLED fiber displays.

Although this work presents the first stretchable fiber OLED display, there remains room for further improvement. First, while this study introduces a novel via strategy to achieve stretchability for the first time, it primarily focuses on realizing omni‐directional stretchability. To enable comprehensive multi‐dimensional mechanical characterization, more advanced strategies, such as incorporating self‐healable or other mechanically adaptive features, will be required in future studies.^[^ ^45^ ^]^ Second, as shown in Figure 5g, the three pixels of the middle fiber OLED exhibit lower luminance compared to those of the other fiber OLEDs. This difference arises from the viewing‐angle limitation of the fiber OLEDs (Figure S7, Supporting Information): the middle fiber OLED was attached with a slight tilt, rather than being perfectly perpendicular to the viewing direction, resulting in a lower apparent luminance. This limitation is attributed to the current fiber OLED structure, in which the thermally evaporated layers are deposited only on one side of the cylindrical fiber surface, leading to directional emission. To overcome this issue, a promising strategy would be to deposit all the layers using a dip‐coating process, enabling the formation of a uniform cylindrical structure that can provide omnidirectional emission. Further optimization will be essential to ensure long‐term stability. Storage lifetime tests conducted on fiber‐based OLEDs showed an LT50 of 17 days (Figure S8, Supporting Information), indicating the need for improved material stability. Future studies incorporating thermally evaporated OLEDs with inherently higher stability could substantially extend their operational lifetime.^[^ ^46^ ^]^

## 3 Conclusion

In this study, the first stretchable fiber‐based OLED display was demonstrated, enabled by a robust, conductive, and deformable via (RCD‐via) that satisfies three critical requirements—electrical conductivity, mechanical deformability, and robust adhesion to scan and data lines. The RCD‐via, composed of silver microparticles embedded in a PDMS matrix, was chemically bonded to metal interconnects through APTES‐assisted siloxane bonding. The novel via structure was precisely designed based on four mechanical boundary conditions and quantitatively verified through FEA. Under a rotational strain of 60°, the von Mises stress within the RCD‐via was simulated to be ≈1.083 MPa, which is less than half of the measured yield strength (2.3 MPa). From a bonding perspective, the simulated maximum stress at the bonding interface was ≈0.176 MPa, corresponding to ≈15% of the measured maximum shear strength of the siloxane bond. Based on precise mechanical design and simulation‐based verification, the effectiveness of the RCD‐via was further validated through experimental results, demonstrating stable performance with minimal variation in resistance (≈2.5%) and luminance (≈7.1%) under 60° of twisting. A 3 × 3 fiber OLED display incorporating a matrix of RCD‐vias was successfully fabricated, exhibiting reliable operation under a stretchability of ≈22.5%. Moreover, after 50 torsion cycles, encapsulated devices maintained stable luminance, indicating that the RCD‐via localized mechanical strain away from the OLED regions, thereby enabling the use of conventional vapor‐deposited encapsulation. By successfully achieving stretchability—a key requirement that must be addressed to realize wearable fiber OLED displays—this work establishes a critical breakthrough toward their practical implementation, providing a scalable and mechanically reliable platform for next‐generation truly wearable fiber OLED displays.

## 4 Experimental Section

### Preparation of Materials

The preparation processes for the OLED materials were similar to those reported in the previous work.^[^ ^25^ ^]^ The PEDOT: PSS formulation was prepared by blending Clevios PH1000 with dimethyl sulfoxide (DMSO) and Zonyl FS‐300 in a weight ratio of 94.5:5:0.5. For the synthesis of ZnO nanoparticles (ZnO NPs), 1.51 g of potassium hydroxide (KOH) was first dissolved in 60 mL of methanol (MeOH), while 3 g of zinc acetate dihydrate (Zn(Ac)_2_·2H_2_O) was separately dissolved in 120 mL of MeOH. Once both solutions were fully dissolved, the KOH solution was added dropwise into the zinc acetate solution under continuous stirring in a round‐bottom flask. The resulting mixture was stirred for 100 min and then subjected to centrifugation at 4000 rpm for 20 min. The ZnO NPs formed in the process were subsequently re‐dispersed in 1‐butanol to obtain a 2 wt.% dispersion. Polyethylenimine (PEI) was diluted using 2‐methoxyethanol to achieve a final concentration of 0.4 wt.%. For the emitting layer (EML), a blend of poly(N‐vinylcarbazole) (PVK), 6‐bis(3‐(9H‐carbazol‐9‐yl)phenyl)pyridine (26DCzppy), and tris(2‐phenylpyridine)iridium(III) (Ir(ppy)_3_) was prepared. The APTES solution was prepared by mixing ethanol (EtOH), deionized water (DI), and APTES in a volume ratio of 90:5:5 (v/v/v), followed by stirring at 300 rpm for 24 h using a magnetic stir bar before use. PDMS was prepared by mixing the base and curing agent at a weight ratio of 10:1 (w/w). PDMS and silver microparticles (Ag MPs) were mixed at 83 wt.% Ag content, and the resulting composite was homogenized using a paste mixer.

### Bonding Process

The O_2_ plasma treatment was performed under the following conditions: 60 W power, 50 sccm oxygen flow rate, and a duration of 1 min. After plasma activation, the sample was immersed in an APTES aqueous solution for 1 min, then removed and thermally treated on a hot plate at 80 °C for 5 min. The treated surface was subsequently brought into physical contact with the opposing interface to initiate bonding.

### Fabrication Process

PET fibers with a diameter of 500 µm were first cleaned through sequential sonication in isopropyl alcohol (IPA) and deionized water (DI water). Following the cleaning process, the fibers were subjected to a layer‐by‐layer dip‐coating procedure using four different solutions: PEDOT: PSS, ZnO nanoparticles (ZnO NPs), polyethylenimine (PEI), and a white emission layer solution. The dip‐coating withdrawal speeds for each solution were 0.7, 2, 10, and 50 mm s^−1^, respectively.^[^ ^25^ ^]^ Each coating step was carried out under ambient pressure within a nitrogen‐filled environment, and followed by thermal annealing at 100 °C for 30 min (PEDOT: PSS), 30 min (ZnO NPs), 10 min (PEI), and 20 min (emission layer), respectively. After solution processing, 4,4,4‐tris(N‐carbazolyl)triphenylamine (TCTA), molybdenum oxide (MoO_3_), and aluminum (Al) were sequentially deposited via thermal evaporation at a base pressure of 3 × 10^−6^ Torr. Fiber OLED fabrication followed previously established procedures reported in the earlier work. To fabricate the 3 × 3 matrix display, the thermally evaporated layers (TCTA, MoO_3_, and Al) were selectively deposited onto three distinct patterned regions on a single fiber, enabling the integration of three individual OLED pixels per fiber. A signal fiber was prepared by thermal evaporation of a 100 nm aluminum (Al) layer onto a polyethylene terephthalate (PET) fiber with a diameter of 500 µm. A 50 nm‐thick Al_2_O_3_ layer was deposited onto the textile display using thermal atomic layer deposition (ALD) at 70 °C for encapsulation purposes. The Al_2_O_3_ film was formed through alternating exposures to trimethylaluminum (TMA) as the aluminum precursor and H_2_O as the oxidizing agent. Subsequently, parylene‐C was deposited at room temperature using a parylene coater (Obang Tech Co.) to provide additional encapsulation. To etch the parylene‐C layer, RIE was performed at 100 W power with an oxygen flow rate of 50 sccm for 3 min, which was sufficient to fully remove the film. For PDMS mold fabrication, patterning was conducted using a CO_2_ laser cutter (Universal Laser VLS3.50 and EPILOG LASER Fusion M2).

### Device Characterization

Cross‐sectional images of the multilayer structure were obtained using a focused ion beam (FIB) system (Quanta 3D FEG, USA) in combination with field‐emission scanning electron microscopy (FE‐SEM, S‐4800, Hitachi Inc.). Electrical and optical performance (*J–V–L* characteristics) was measured using a source meter (2400 Series, Keithley Inc.) and a spectroscopic radiometer (CS2000, Konica Minolta Inc.) equipped with a close‐up lens (CS‐A35). Cyclic bending tests were conducted using a mechanical bending test system (Sciencetown Inc.). XPS depth profiling was performed using a K‐Alpha⁺ XPS system (Thermo Fisher Scientific) equipped with a 200 µm X‐ray spot size. Finite element‐based mechanical simulations were performed using ANSYS software. PDMS patterning was performed using a CO_2_ laser cutter (Universal Laser VLS3.50 and EPILOG LASER Fusion M2).

### Statistical Analysis

The output performance—including luminance, current, and voltage—was obtained directly from measurements using the instruments described above. The material property data and analyses, such as those shown in Figures 2, 4,5h,j,g, were based on 3–5 repeated measurements, and the results were presented with error bars. All curves were plotted using Origin software. Normalized data were scaled relative to the maximum value of each dataset.

## Conflict of Interest

The authors declare no conflict of Interest.

## Supporting information



Supporting Information

Supplemental Movie 1

Supplemental Movie 1

## Data Availability

The data that support the findings of this study are available from the corresponding author upon reasonable request.
