# Supplementary material for: Stretchable Fiber‐Based Organic Light‐Emitting Diodes Display Enabled by Robust, Conductive, and Deformable Via
Source: Adv Sci (Weinh). 2025 Oct 7;12(48):e14594. doi: 10.1002/advs.202514594 (PMC12752599; doi:10.1002/advs.202514594)
Supplement: Supplementary file 1 — Supporting Information [file ADVS-12-e14594-s001.docx]

Supplementary Information

**Stretchable Fiber-based Organic Light-Emitting Diodes enabled by Robust, Conductive, and Deformable Via**

Yong Ha Hwang, Hagseon kim, and Kyung Cheol Choi*

Y. H. Hwang, H. Kim, Prof. K. C. Choi

School of Electrical Engineering,

Korea Advanced Institute of Science and Technology,

Daejeon 34141, Republic of Korea.

*E-mail: kyungcc@kaist.ac.kr

**Table S1.** Comparison of previously reported fiber-based light-emitting devices

| Device type | Source | Fiber type | Array | Interconnection |
| --- | --- | --- | --- | --- |
| Display | ACEL[1] | Cylinder | No | No |
|  | ACEL[2] | Cylinder | Yes | No |
|  | OLED[3] | Flat Rectangular | Yes | No |
|  | OLED[4] | Flat Rectangular | Yes | No |
|  | OLED[5] | Cylinder | No | No |
|  | OLED[6] | Cylinder | No | No |
|  | OLED[7] | Cylinder | No | No |
|  | OLED[8] | Cylinder | No | No |
|  | OLED[9] | Cylinder | No | No |
|  | OLED[10] | Cylinder | Yes | No |
|  | OLED[11] | Cylinder | No | No |
|  | OLED[12] | Cylinder | No | No |
|  | OLED[13] | Cylinder | No | No |
|  | OLED[14] | Cylinder | Yes | Rigid  (silver paste) |
|  | QLED[15] | Flat Rectangular | Yes | Rigid  (silver paste) |
|  | LED[16] | Flat Rectangular | X-axis | - |
|  | OLED  [This work] | Cylinder | Yes | Flexible  (C-PDMS) |

**Supporting information references**

[1] Z. Zhang, L. Cui, X. Shi, X. Tian, D. Wang, C. Gu, E. Chen, X. Cheng, Y. Xu, Y. Hu, J. Zhang, L. Zhou, H. H. Fong, P. Ma, G. Jiang, X. Sun, B. Zhang, H. Peng, *Adv. Mater.* 2018, *30*, 1800323.

[2] Shi, X.; Zuo, Y.; Zhai, P.; Shen, J.; Yang, Y.; Gao, Z.; et al. "Large-Area Display Textiles Integrated with Functional Systems". Nature 2021, 591 (7849), 240–245.

[3] Song, Y. J.; Kim, J.-W.; Cho, H.-E.; Son, Y. H.; Lee, M. H.; Lee, J.; Choi, K. C.; Lee, S.-M. Fibertronic Organic Light-Emitting Diodes toward Fully Addressable, Environmentally Robust, Wearable Displays. ACS Nano 2020, 14 (1), 1133–1140.

[4] Song, H.; Song, Y. J.; Hong, J.; Kang, K. S.; Yu, S.; Cho, H. E.; Kim, J. H.; Lee, S. M. Water Stable and Matrix Addressable OLED Fiber Textiles for Wearable Displays with Large Emission Area. npj Flexible Electronics 2022, 6 (1), 1–8.

[5] O’Connor, B.; An, K. H.; Zhao, Y.; Pipe, K. P.; Shtein, M. Fiber Shaped Light Emitting Device. Advanced Materials 2007, 19 (22), 3897–3900.

[6] Ko, K.-J.; Lee, H. B.; Kim, H. M.; Lee, G. J.; Shin, S.-R.; Kumar, N.; Song, Y. M.; Kang, J.-W. High-Performance, Color-Tunable Fiber Shaped Organic Light-Emitting Diodes. Nanoscale 2018, 10 (34), 16184–16192.

[7] Kong, B.K., Kim, D.H. and Kim, T.W.,Significant enhancement of out-coupling efficiency for yarn-based organic light-emitting devices with an organic scattering layer. Nano Energy 2020, 70

[8] Kwon, S.; Kim, W.; Kim, H.; Choi, S.; Park, B.-C.; Kang, S.-H.; Choi, K. C. High Luminance Fiber-Based Polymer Light-Emitting Devices by a Dip-Coating Method. Adv Electron Mater 2015, 1 (9), 1500103

[9] Kwon, S.; Kim, H.; Choi, S.; Jeong, E. G.; Kim, D.; Lee, S.; Lee, H. S.; Seo, Y. C.; Choi, K. C. Weavable and Highly Efficient Organic Light-Emitting Fibers for Wearable Electronics: A Scalable, Low-Temperature Process. Nano Lett 2018, 18 (1), 347–356.

[10] Hwang, Y. H.; Kwon, S.; Shin, J. Bin; Kim, H.; Son, Y. H.; Lee, H. S.; Noh, B.; Nam, M.; Choi, K. C. Bright-Multicolor, Highly Efficient, and Addressable Phosphorescent Organic Light-Emitting Fibers: Toward Wearable Textile Information Displays. Adv Funct Mater 2021, 2009336, 1–10.

[11] Hwang, Y. H.; Noh, B.; Lee, J.; Lee, H. S.; Park, Y.; Choi, K. C. High‐Performance and Reliable White Organic Light‐Emitting Fibers for Truly Wearable Textile Displays. Advanced Science 2022, 2104855, 2104855.

[12] Kong, S. U.; Jeon, Y.; Lee, H. S.; Hwang, Y. H.; Chang, J.; Kim, H.; Kim, C. Y.; Choi, K. C. Anode-Patterned Monorail-Structure Fiber-Based Organic Light-Emitting Diodes with Long Lifetime and High Performance for Truly Wearable Displays. Adv Opt Mater 2023, 2203130, 1–9.

[13] Kim, C. Y.; Hwang, Y. H.; Chang, J.; Kong, S. U.; Park, S.-H. K.; Choi, K. C. High Mobility, Low Off-Current, and Flexible Fiber-Based a-InGaZnO Thin-Film Transistors toward Wearable Textile OLED Displays. ACS Appl Mater Interfaces 2024, 16 (45), 62335–62346.

[14] Hwang, Y. H., Kim, C. Y., & Choi, K. C. Three-Dimensional Connective Architecture-Based Integrated Organic Light-Emitting Diodes and Transistors on Fiber for Drive Circuitry-Enabled Fiber Displays. ACS Nano 2025, 19, 27, 25515–25525

[15] Lee, S.; Choi, H. W.; Figueiredo, C. L.; Shin, D.; Moncunill, F. M.; Ullrich, K.; et al. "Truly Form-Factor – Free Industrially Scalable System Integration for Electronic Textile Architectures with Multifunctional Fiber Devices". Science Advances 2023, No. i, 1–11.

[16] Choi, H. W.; Shin, D.-W.; Yang, J.; Lee, S.; Figueiredo, C.; Sinopoli, S.; et al. "Smart Textile Lighting/Display System with Multifunctional Fibre Devices for Large Scale Smart Home and IoT Applications". Nature Communications 2022, 13 (1), 814.


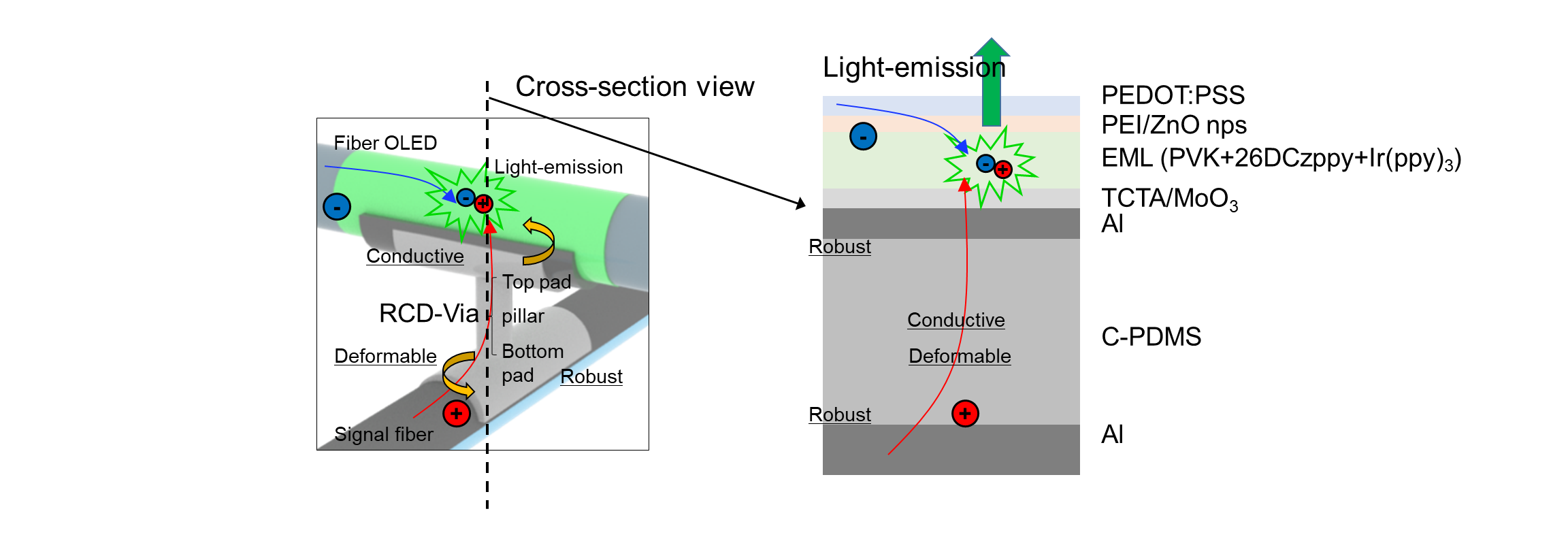


**Figure S1.** Holes and electrons are injected from the signal and data lines, respectively, and recombine in the emissive layer to emit light from the top of the fiber OLED. The cross-sectional images illustrate the pathways of charge carriers as they travel through the OLED structure.


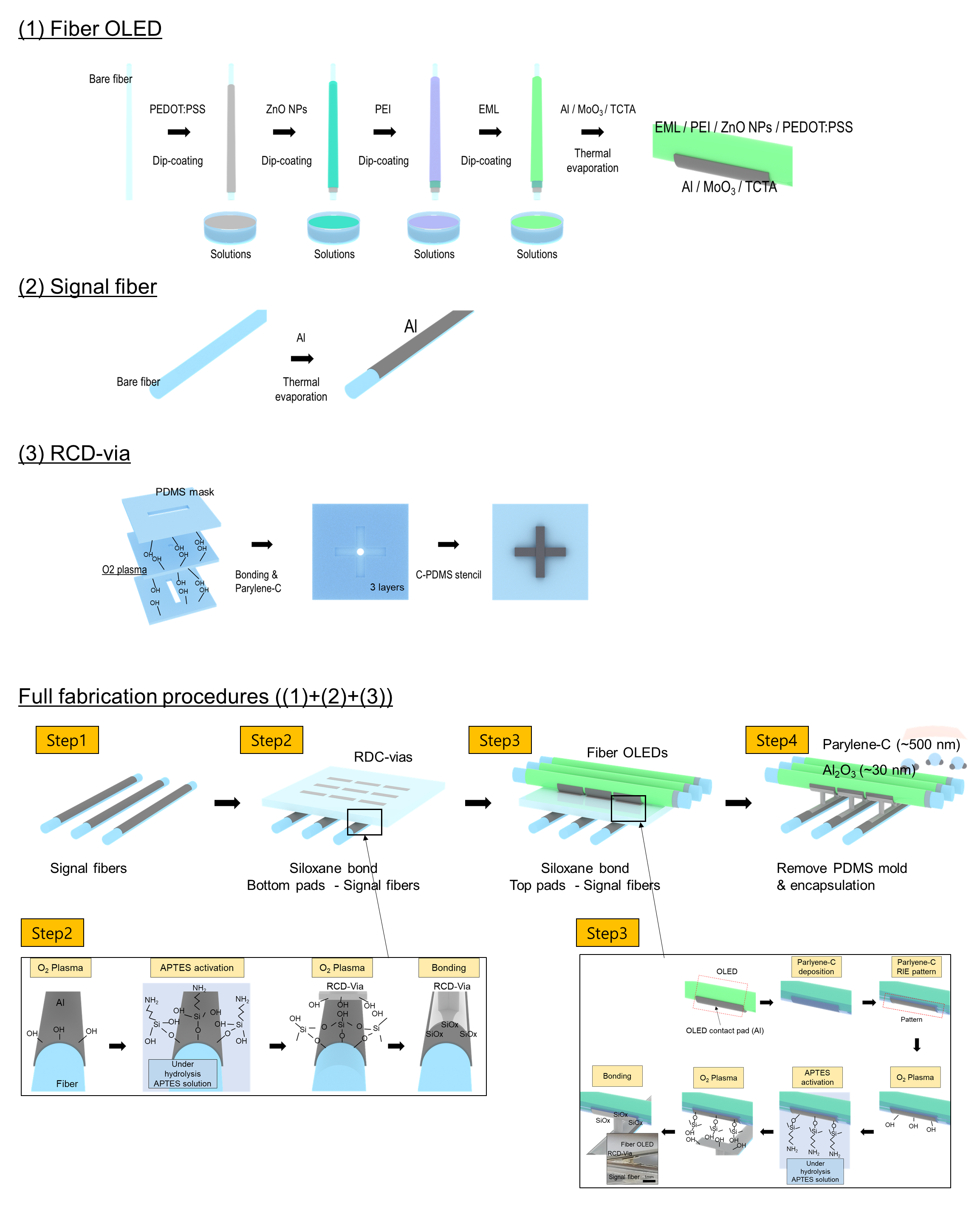
 **Figure S2.** Full fabrication procedure schematic for the stretchable fiber display. The schematic illustrates the fabrication processes of each component — the fiber OLED, the signal fiber, and the RCD-via — and presents the step-by-step integration of these components into the final stretchable fiber display.


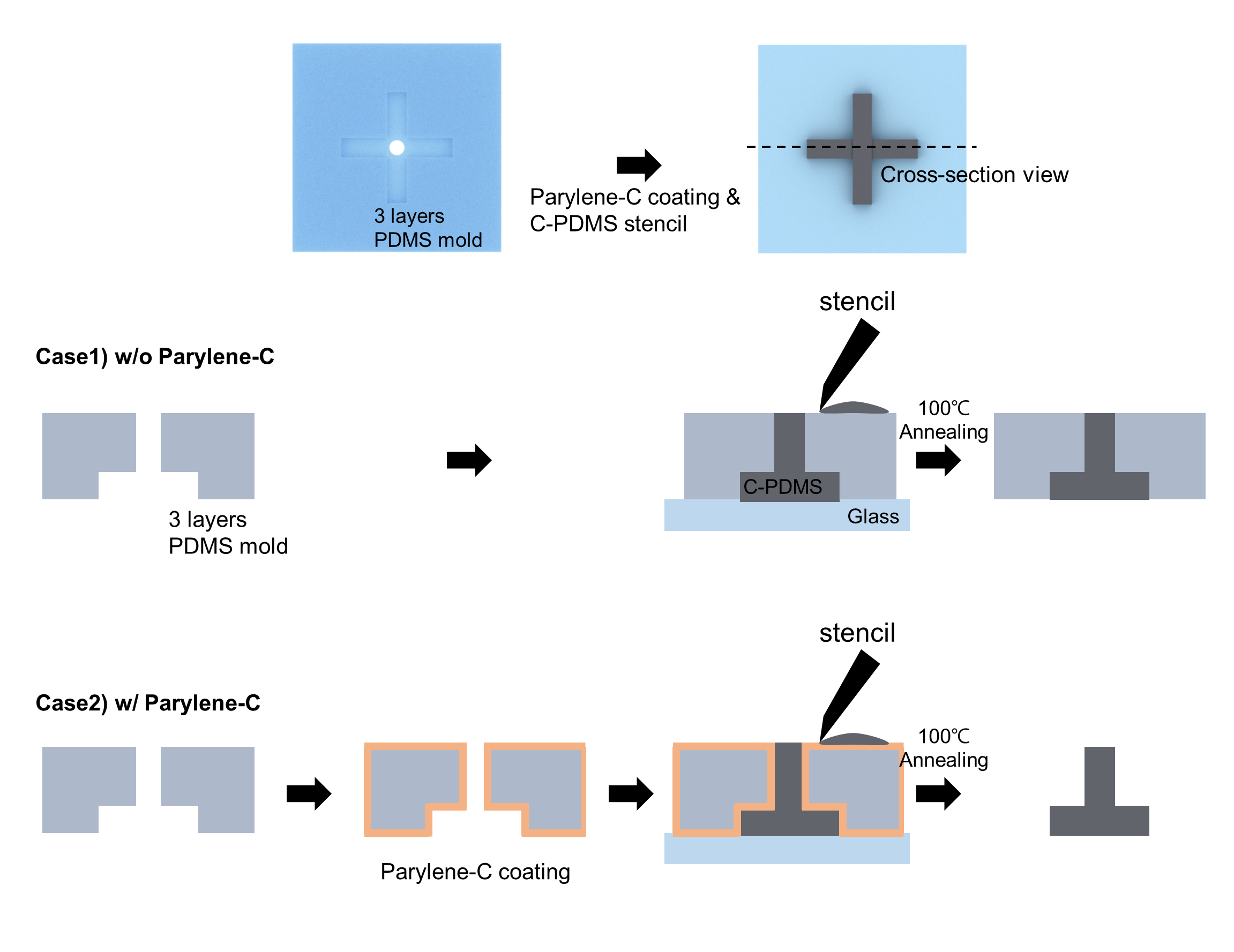
 **Figure S3.** **Cross-sectional schematic of the RCD-via fabrication process using stencil filling of C-PDMS into a parylene-C-coated PDMS mold.**

The schematic illustrates the fabrication steps from a cross-sectional view, including parylene-C deposition onto the PDMS mold, stencil application of C-PDMS, and demolding after thermal curing. Two cases are compared: **Case 1** (without parylene-C coating) shows that C-PDMS irreversibly bonds with the PDMS mold during curing, making demolding impossible. **Case 2** (with parylene-C coating) demonstrates that the parylene-C layer acts as an anti-adhesive barrier, preventing undesired crosslinking and enabling clean release of the RCD-via post-annealing.


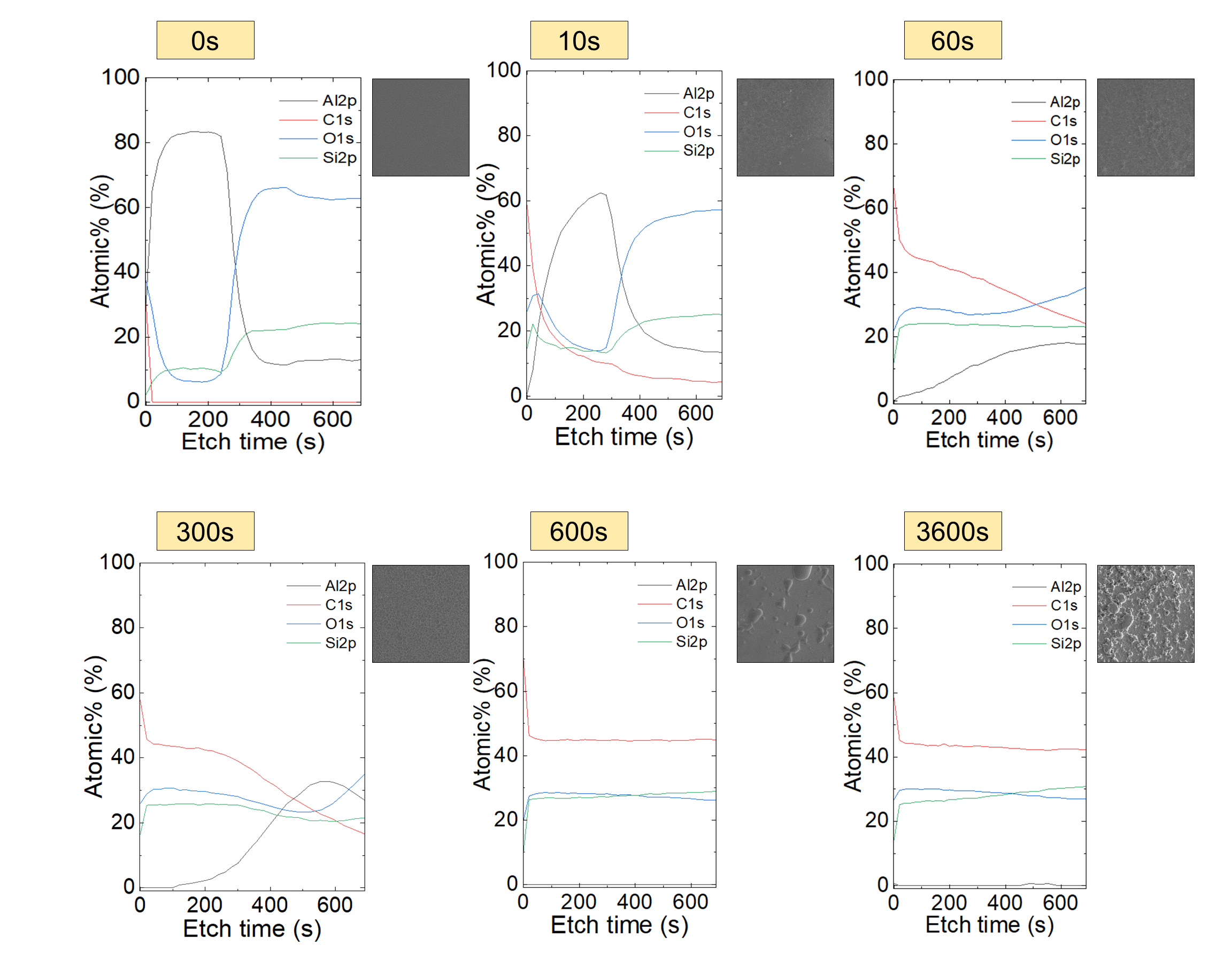


**Figure S4.** **XPS depth profile and SEM surface analysis of Al substrates treated with various APTES activation times.**

XPS depth profiles for activation times of 0 s, 10 s, 60 s, 300 s, 600 s, and 3600 s show a gradual decrease in Al signal intensity at the surface with increasing treatment time, indicating progressive accumulation of SiOₓ and byproducts. This surface evolution is further corroborated by SEM images, which reveal increased surface roughness and coverage with longer activation durations.


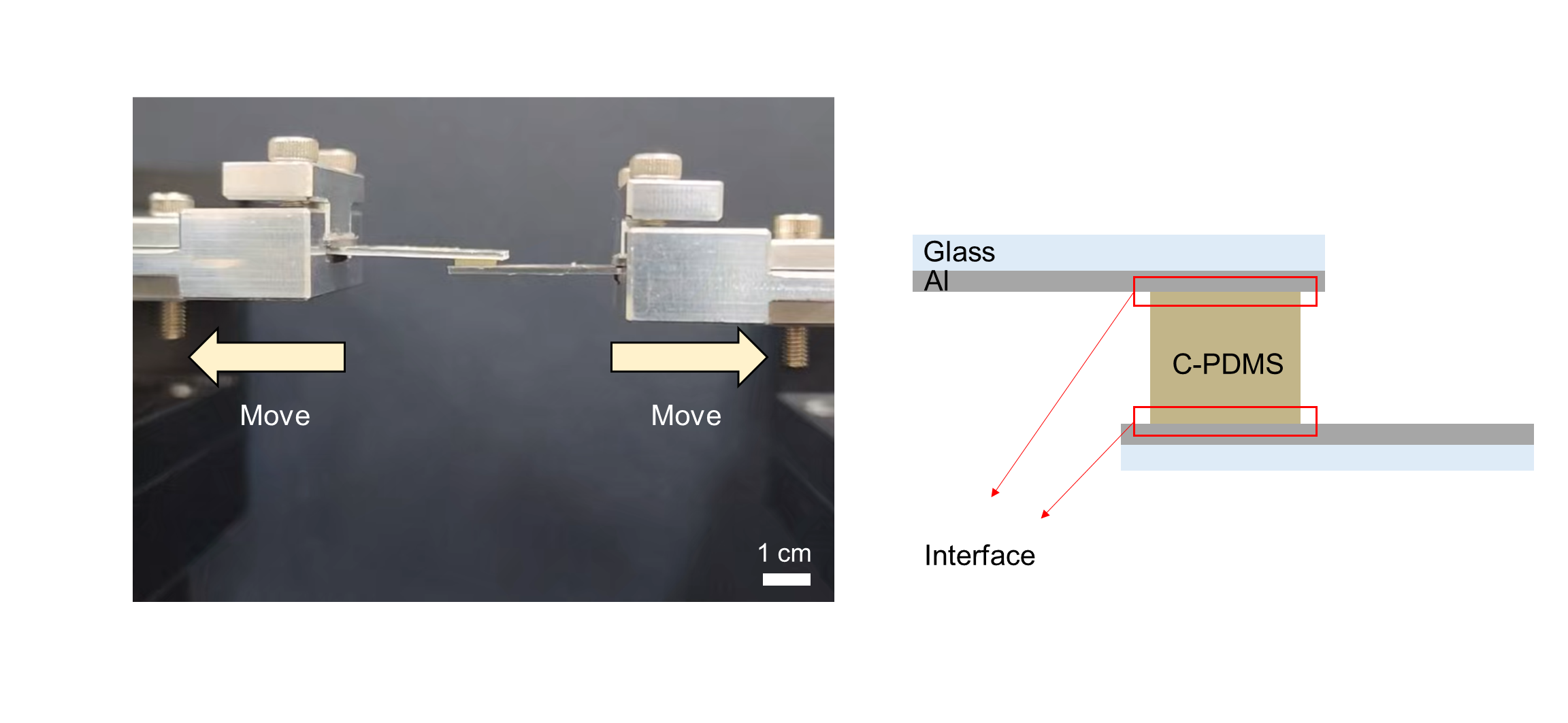


**Figure S5.** **Fracture shear test setup and schematic illustration of bonding interface evaluation.**

The left photograph shows the tensile testing machine and sample mounted for fracture shear testing, while the right schematic illustrates the test sample configuration. The bonding strength at the interface was evaluated for various APTES activation times, as well as for control samples using commercial superglue and adhesive tape. During testing, the tensile machine applies lateral displacement to both ends of the sample, generating shear stress at the bonding interface. The applied force is measured via a load cell integrated into the testing system.


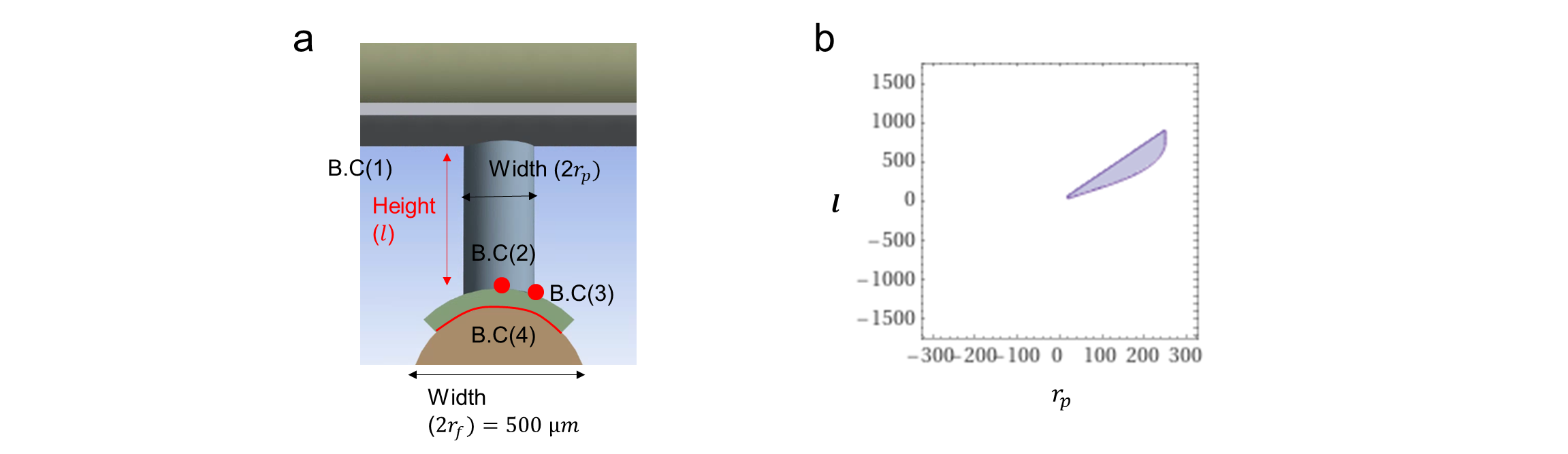


**Figure S6.** (a) Four boundary conditions (b) Governing equation (3) solutions.

Governing equations

$\frac{2l}{r_{p}}< \sqrt{\frac{\pi^{2}E}{\text{σy }}}$ ··· (1)

$\frac{E}{\left. 2(1+v \right)}* \frac{r_{p}\theta}{\sigma_{y}}<l$ ··· (2)

$\sqrt{\left( E*\frac{r_{f} - \sqrt{{r_{f}}^{2}-{r_{p}}^{2}}}{l} \right)^{2}+4\left( \frac{E}{\left. 2(1+v \right)}* \frac{r_{p}\theta}{l+r_{f} - \sqrt{{r_{f}}^{2}-{r_{p}}^{2}}} \right)^{2}}<\sigma_{y}$ ··· (3)

$r_{p}=\sqrt{\left( r_{f} \right)^{2}-\left( r_{f}-2r_{f}\left( \frac{KH}{E} \right)^{2} \right)^{2}}$ ··· (4)

Solution of governing equation

$$\theta=\frac{\pi}{3}, E=7.122 MPa , \sigma_{y}=2.3 MPa, H=2.23 MPa$$

$$From Equation (1)$$

$$\frac{2l}{r_{p}}<\sqrt{\frac{{2\pi}^{2}E}{\text{σ}\text{y }}}= \sqrt{\frac{{2\pi}^{2}*7.122MPa}{2.3MPa}}=7.818$$

$\therefore l<3.909r_{p}$, $r_{p}<250 (Because Radius of fiber=250um)$

$$From Equation (2)$$

$\frac{E}{2(1+v)}* \frac{r_{p}\theta}{\sigma_{y}}<l$ , $\therefore1.088r_{p}<l$

$$\therefore1.088r_{p}<l<3.909r_{p}$$

$$From Equation (4)$$

$$\boldsymbol{\therefore}\boldsymbol{r}_{\boldsymbol{p}}=\sqrt{\left( 250 \right)^{2}-\left( 250-2*250*\left( \frac{0.6549*2.23}{7.122} \right)^{2} \right)^{2}}=\boldsymbol{100.35 um}$$

$$From Equation \left( 1 \right), (2)$$

$$\boldsymbol{\therefore208.5<l<392.2}\boldsymbol{um}$$

$$From Equation (3)$$

$$\therefore\sqrt{\left( 7.122*\frac{250 - \sqrt{{250}^{2}-{r_{p}}^{2}}}{l} \right)^{2}+\left( 5.005* \frac{r_{p}}{l+250 - \sqrt{{250}^{2}-{r_{p}}^{2}}} \right)^{2}}<5.29$$

A pillar height of 390 μm was selected, as greater height reduces torsional stress exerted on the structure. When the pillar radius (rₚ) is set to 100 μm, this height also satisfies the condition defined in equation (3), validating the selection from both mechanical and geometric perspectives.


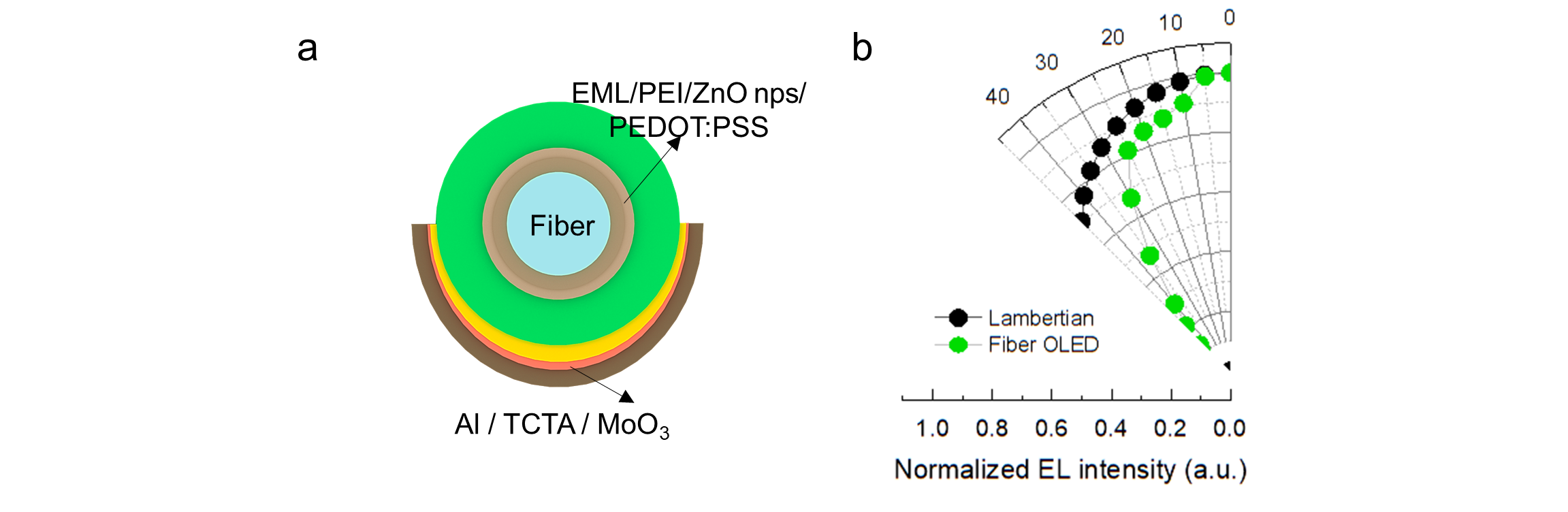
 **Figure S7.** (a) Cross-sectional structure of the fiber OLED. (b) Luminance of the fiber OLED as a function of viewing angle.

Since OLEDs are planar light sources, conventional planar OLEDs exhibit Lambertian characteristics, with the intensity varying approximately as cos θ with viewing angle. In contrast, fiber OLEDs have a cylindrical geometry, resulting in a relatively higher central intensity compared to planar devices.


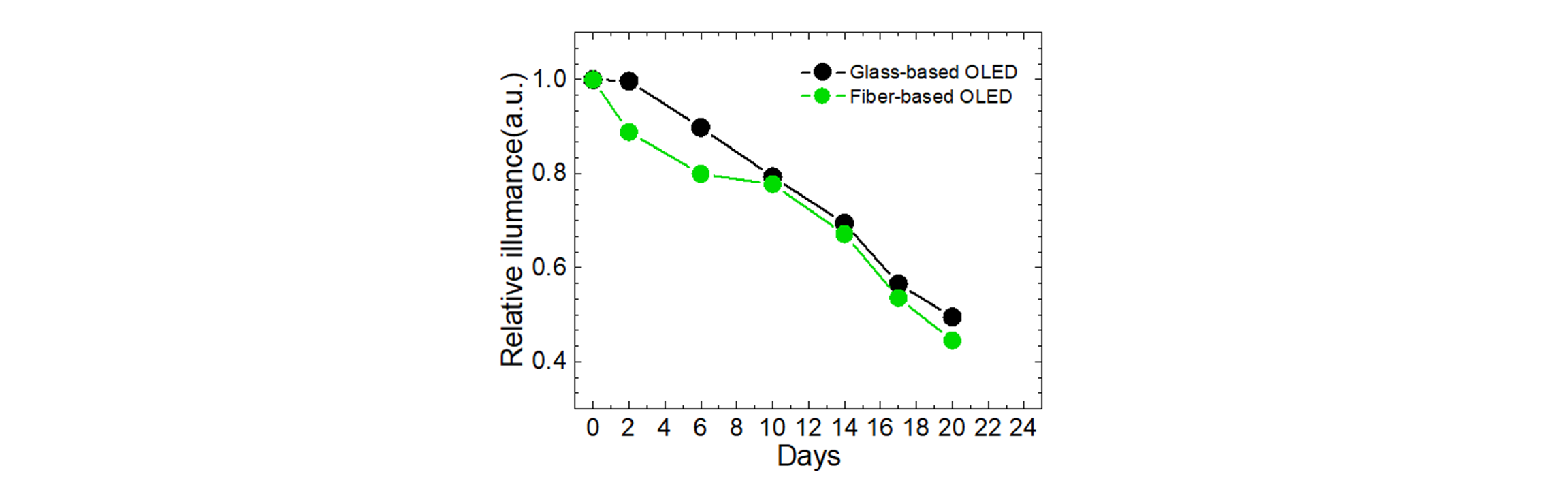
 **Figure S8. Comparison of storage lifetime between fiber-based and glass-based OLEDs with identical stack structures.**

**Both devices exhibited similar degradation trends, reaching LT₅₀ after 17 days, indicating comparable operational stability.** This comparable degradation behavior indicates that the limited operational lifetime is not attributed to the proposed 3D fiber-based stack-up, as the glass-based OLEDs with the same stack-up exhibited similar degradation characteristics. The degradation is likely attributed to the main factor that is the inherently shorter lifetime of solution-processed OLEDs.


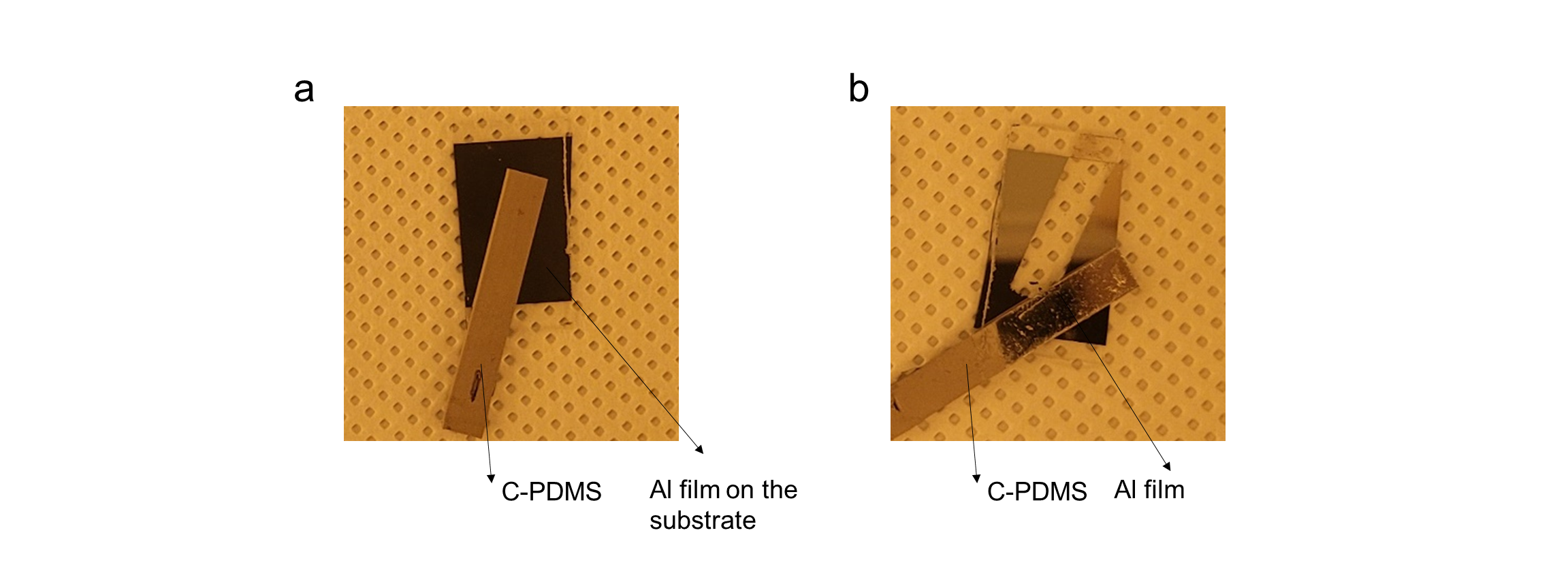


**Figure S9**. (a) C-PDMS chemically bonded to the Al film deposited on the substrate via APTES treatment.(b) After peel-off, the Al thin film remains attached to the C-PDMS, indicating that separation occurred between the substrate and the Al layer.

The peel-off test shows that when the C-PDMS chemically bonded to the Al is peeled off, the separation occurs between the substrate and the Al layer, with the Al thin film remaining attached to the C-PDMS.


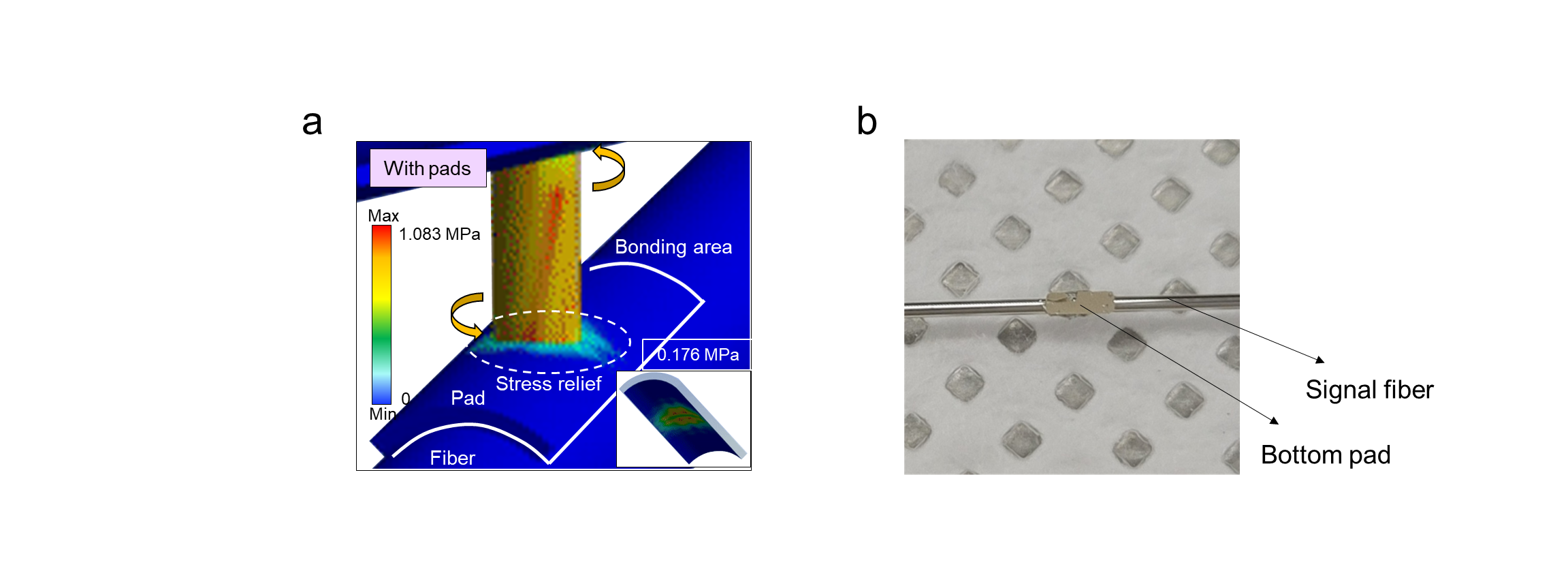


**Figure S10.** (a) Simulated stress distribution highlighting the mechanically vulnerable region of the RCD-via. (b) Under strong applied strain, the pad remains on the Al layer while the pillar becomes detached.

From a device perspective, the RCD-via has a cross-bar-shaped pad structure, and the region where the pad connects to the pillar is mechanically the most vulnerable under strain, as demonstrated by the fact that strong mechanical stress causes separation between the pad and the pillar.


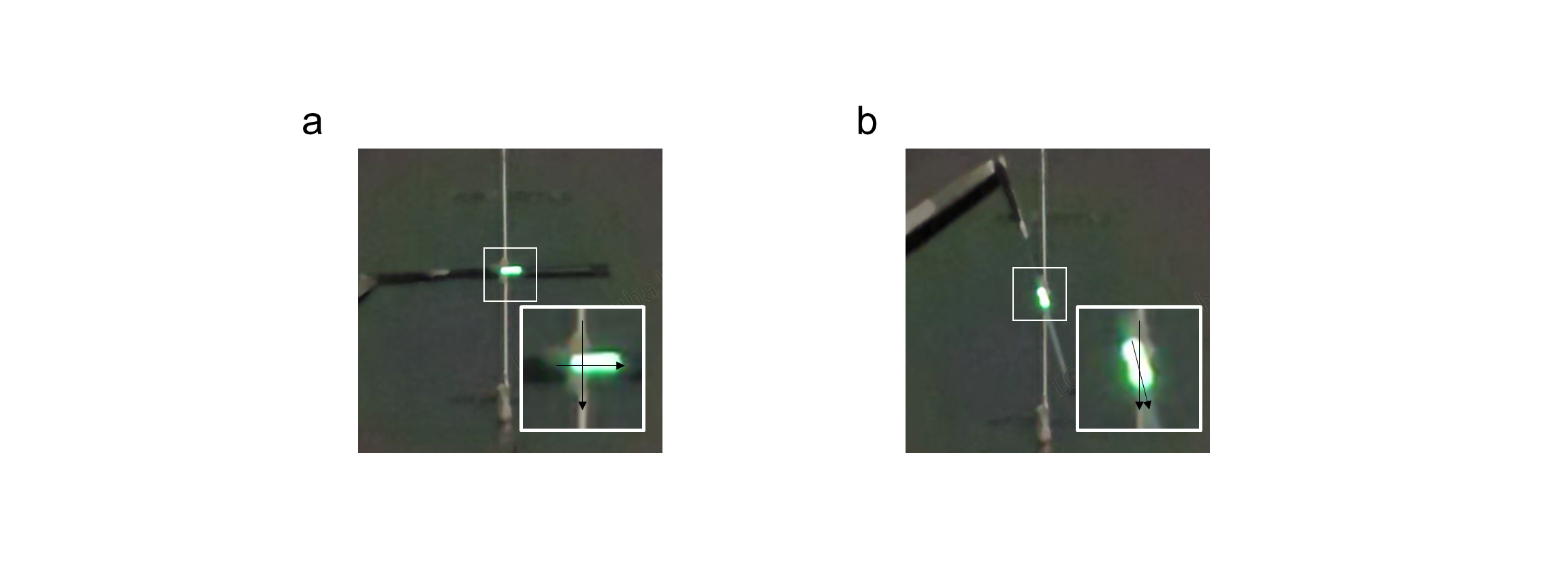


**Figure S11.** (a) Initial state (0° rotation) of a single fiber OLED with a single RCD-via connecting it to a signal fiber. (b) Fiber OLED operating reliably even when the single RCD-via is rotated nearly 90°.

By applying the governing equation (2) inversely using the pillar radius (100 μm) and height (~400 μm) obtained from the governing equation in Figure 4, the maximum possible rotation angle of the single RCD-via is calculated to be approximately 220°.

$\frac{E}{2(1+v)}* \frac{r_{p}\theta}{\sigma_{y}} <l$ ··· (2)

$\frac{7.122}{2*1.49}* \frac{100*\theta}{2.3} <400$,

∴ θ$<1.22\pi$ = 220°

Since the most commonly experienced rotation of an interconnector in an x–y array is ideally around ±90°, this result demonstrates that the proposed RCD-via can withstand even beyond typical extreme conditions. Notably, an array requires smaller rotation angles than a single via. Experimental results further confirm that even a single via operates reliably at nearly 90° of rotation, indicating that the RCD-via provides sufficient mechanical durability for practical implementation in stretchable fiber OLED arrays.


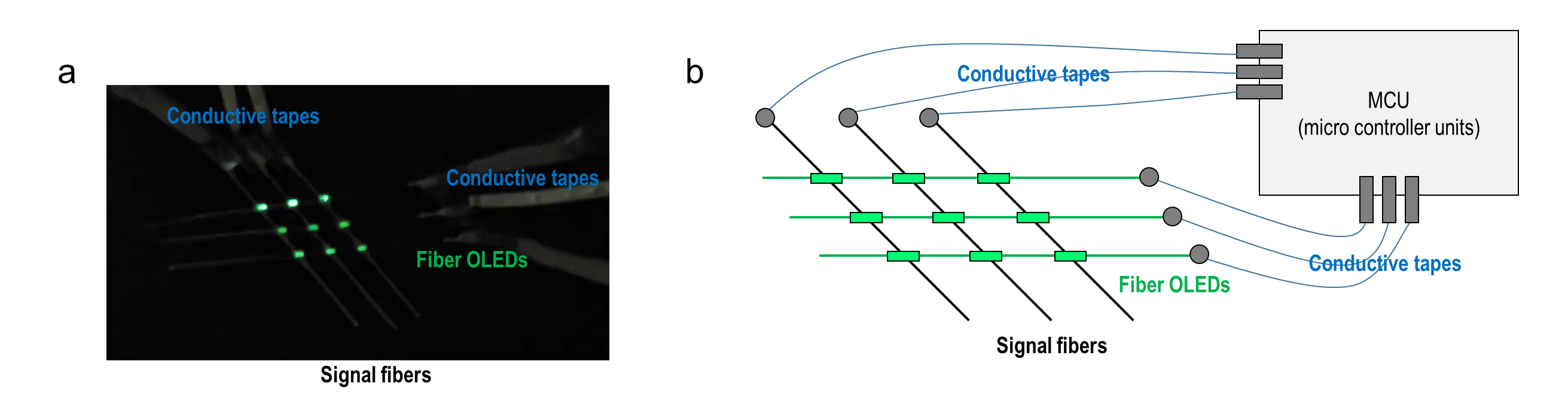


**Figure S12.** (a) Photograph of a 3×3 stretchable fiber OLED display with its six lines each connected to conductive tape. (b) Schematic illustration showing the six lines of the 3×3 stretchable fiber OLED display connected to conductive tape, which is then interfaced with the MCU..
